# Development of stroke predictive model in community-dwelling population: A longitudinal cohort study in Southeast China

**DOI:** 10.3389/fnagi.2022.1036215

**Published:** 2022-12-22

**Authors:** Qi Wang, Lulu Zhang, Yidan Li, Xiang Tang, Ye Yao, Qi Fang

**Affiliations:** ^1^Department of Biostatistics, School of Public Health, Fudan University, Shanghai, China; ^2^Department of Neurology, First Affiliated Hospital of Soochow University, Suzhou, China; ^3^National Clinical Research Centre for Aging and Medicine, Huashan Hospital, Fudan University, Shanghai, China; ^4^Key Laboratory of Public Health Safety of Ministry of Education, Fudan University, Shanghai, China

**Keywords:** stroke, risk, factors, predictive model, ROC analysis

## Abstract

**Background:**

Stroke has been the leading cause of death and disability in the world. Early recognition and treatment of stroke could effectively limit brain damage and vastly improve outcomes. This study aims to develop a highly accurate prediction model of stroke with a list of lifestyle behaviors and clinical characteristics to distinguish high-risk groups in the community-dwelling population.

**Methods:**

Participants in this longitudinal cohort study came from the community-dwelling population in Suzhou between November 2018 and June 2019. A total of 4,503 residents participated in the study, while stroke happened to 22 participants in the 2-year follow-up period. Baseline information of each participant was acquired and enrolled in this study. *T*-test, Chi-square test, and Fisher’s exact test were used to examine the relationship of these indexes with stroke, and a prediction scale was constructed by multivariate logistic regression afterward. Receiver operating characteristic analysis was applied to testify to the prediction accuracy.

**Results:**

A highly accurate prediction model of stroke was constructed by age, gender, exercise, meat and vegetarian diet, BMI, waist circumference, systolic blood pressure, Chinese visceral adiposity index, and waist-height ratio. Two additional prediction models for overweight and non-overweight individuals were formulated based on crucial risk factors, respectively. The stroke risk prediction models for community-dwelling and overweight populations had accuracies of 0.79 and 0.82, severally. Gender and exercise were significant predictors (χ^2^ > 4.57, *p* < 0.05) in the community-dwelling population model, while homocysteine (χ^2^ = 4.95, *p* < 0.05) was significant in the overweight population model.

**Conclusion:**

The predictive models could predict 2-year stroke with high accuracy. The models provided an effective tool for identifying high-risk groups and supplied guidance for improving prevention and treatment strategies in community-dwelling population.

## Introduction

Stroke remains high in the global disease burden for a long time (Gorelick, 2019). It is the leading cause of death and disability in the world (Diseases and Injuries, 2020). There were 12.2 million stroke events in 2019, with ischemic stroke accounting for 62.4% of all, cerebral hemorrhage at 27.9%, and subarachnoid hemorrhage at 9.7% (Collaborators, 2021). According to the Global Burden of Disease Study 2019, there were 3.9 million new stroke cases in China, and the incidence of stroke increased by 86.0% from 1990 to 2019 (Ma et al., 2021). The number of Chinese adults dying or disabled due to stroke has been increasing, but fortunately, lifestyle interventions and early drug treatment have been shown to be effective in reducing stroke morbidity and mortality (Caldwell et al., 2019; Chao et al., 2021). Early treatment of ischemic stroke with intravenous tPA could reduce the risk of disability (Mitka, 2013).

Visceral adipose tissue could surround organs and deposit in blood vessels, producing various adipokines and hormones. The accumulation of visceral fat could lead to endocrine and metabolic comorbidities (Aparecida Silveira et al., 2020). It may be associated with increased adipocytokine production, pro-inflammatory activity, and altered lipid levels, and decreased high-density lipoprotein (HDL) cholesterol (Tchernof and Despres, 2013). The visceral adiposity index (VAI) and Chinese visceral adiposity index (CVAI) were effective in assessing visceral adiposity and significantly associated with cardiovascular and cerebrovascular events (Amato et al., 2010; Xia et al., 2016). The indexes may be of great value in predicting stroke.

Common risk factors for stroke have been identified by several studies. Elevated systolic blood pressure and total cholesterol increased stroke risk, and the effect varied by gender, with men being more sensitive (Jee et al., 2008). In various types of hypertensions, elevated systolic blood pressure was the most common and more difficult to control. Different from diastolic blood pressure, systolic blood pressure gradually increased with age, which has greater clinical significance (Strandberg and Pitkala, 2003). High systolic blood pressure has always been an important risk factor of stroke burden, which was positively related to the occurrence and development of stroke. According to previous studies, the increase of systolic blood pressure would lead to the thickening of arterial wall and narrowing of lumen, enhance endothelial dysfunction and vascular permeability, cause local thrombosis, and also promote the development of atherosclerosis, forming vascular resistance (Ma et al., 2022). Most previous studies have indicated that high total cholesterol increased the risk of ischemic stroke, which may be related to high total cholesterol leading to atherosclerosis (Gu et al., 2019). Overweight was associated with a high risk of stroke, possibly independent of other vascular risk factors (Strazzullo et al., 2010). Negative lifestyle behaviors, such as smoking, alcohol consumption, and lack of exercise, were conducive to stroke and its subtypes (Qi et al., 2020). Lack of physical activity was an independent risk factor for stroke, which might be attributed to the increase in energy expenditure and the reduction of blood pressure and lipids through exercise. Based on these factors, previous studies constructed various risk prediction models for stroke and its subtypes, while most of these models consisted of clinical characteristics and rarely involved lifestyle behaviors (Chien et al., 2010; Xing et al., 2019; Arafa et al., 2021).

In this connection, our study aimed to identify a series of lifestyle behaviors and clinical indicators related to stroke, and to construct a prediction model in community-dwelling population based on these potential risk factors. Based on previous findings suggesting that overweight individuals had a higher risk of stroke, we formulated models for overweight and non-overweight individuals, respectively. There is no effective cure for stroke at present, but lifestyle interventions and early treatment could effectively limit brain damage and improve outcomes in high-risk groups. Therefore, early identification and intervention have become the keys. This study aimed to distinguish high-risk groups with high accuracy based on the models and provide evidence support for early treatment.

## Materials and methods

### Study design and participants

The study participants were from the community-dwelling population in Suzhou between November 2018 and June 2019, who had lived in the local area for a certain period (in the past 12 months, had lived in the area for 6 months and more). We selected two communities that are similar to the national situation in terms of social and economic development, population age, and gender composition as the survey areas, and completed the survey for the qualified residents in these two communities. The proportion of those who cannot complete the investigation due to special circumstances (such as refusal of investigation) was less than 15%. We recruited 4,503 participants over 40 years old (including a very small number of residents under 40 years old; Supplementary Figure I). The inclusion criteria were all permanent residents of these two communities, who did not violate their personal wishes and sign the informed consent form. Those with the following conditions had been excluded: (1) preexisting stroke; (2) brain tumor; (3) end-stage critical illness; (4) abnormal mental state; and (5) other diseases that cannot cooperate with the completion of information collection. We followed up with all participants for 2 years, and confirmed their outcomes by searching and matching the Suzhou Center for Disease Control and Prevention database. All participants were divided into patient and control groups according to stroke. We separated the study population into two subgroups based on BMI. Participants with a BMI of 24 or more were considered overweight and were grouped together. Those with a BMI of less than 24 were grouped into another group.

Basic information on each participant who met the inclusion and exclusion criteria was collected, including demographic information, contact details, and status at the time of this survey. The lifestyle behavior, medical history, and physical examinations of participants were investigated and taken by community doctors. Venous blood was collected from participants who fasted for 8 h. The data of laboratory-related indicators were obtained by medical staff through rapid detection in the laboratory. All the above investigations were completed in the community hospital where the participants were located, and the questionnaires were filled out by community doctors. All staff were uniformly trained by relevant experts.

This study was approved by the Ethics Committee of First Affiliated Hospital of Soochow University (No. 2022209). We obtained the informed consent of all participants.

### Vascular risk factors and other co-variates

We collected the following variables: age, gender, lifestyle behaviors (including smoking, drinking, dietary, and exercise), family history of disease (including stroke, coronary heart disease, hypertension, and diabetes), personal medical history and control (including cerebrovascular disease, heart disease, hypertension, dyslipidemia, and diabetes), BMI, waist circumference, blood pressure, heart murmur, heart rhythm, related laboratory indicators, VAI, CVAI, triglyceride-glucose index, and waist-height ratio.

Both systolic and diastolic blood pressure values were the average of two blood pressure measurements. The triglyceride-glucose index (Simental-Mendia et al., 2008), waist-height ratio (Ashwell et al., 1996), VAI (Amato et al., 2010), and CVAI (Xia et al., 2016) were calculated according to established formulas.

### Existing stroke risk score

In 2012, the Stroke Prevention and Control Engineering Committee of the China Health and Family Planning Commission proposed the “8 + 2” stroke risk score (China, 2012). According to the scoring criteria, we could assess the risk of each research participant based on their disease history and information from this survey.

### Statistical tests

Baseline characteristics of all participants were statistically described, and they were divided into two groups according to the occurrence of stroke. The demographic and clinical characteristics of the two groups were compared. Continuous variables were expressed as mean ± standard deviation and were statistically examined using two independent samples *T*-test; categorical data were statistically examined using Chi-square test and Fisher’s exact test, and a two-sided *p*-value was given. Multiple logistic regression model was utilized to perform multivariate analysis and construct a stroke risk prediction scale. We appropriately relaxed the threshold of *p*-value for univariate analysis and considered statistically significant variables for multivariate analysis. In addition, we separately analyzed the related factors of stroke in overweight and non-overweight groups, and constructed corresponding risk prediction scales. The stroke prediction scale of the subgroup population was constructed using a method similar to that of the whole population, that is, univariate analysis was conducted first, and then, age, gender, and significant influencing factors were included in the multivariate model. Receiver operating characteristic (ROC) analysis was applied to testify to the accuracy of the predictive scales. The Youden index and McNemar’s test were exploited to compare the prediction effect with the “8 + 2” stroke risk score. The *p* < 0.05 was considered statistically significant. The data were organized and analyzed using SAS 9.4 and R 4.2.0 software.

## Results

In this study, 4,503 participants who met the inclusion criteria were included, with an average age of 58 years. All participants came from two communities, 2,768 (61.47%) from Xinghai Community and 1735 (38.53%) from Bailian Community (Table 1). Among the participants from the two communities, 952 (34.39%) and 605 (34.87%) were male, respectively. There was no significant difference in gender distribution between the participants of the two communities (χ^2^ = 0.74, *p* > 0.05). There were 1,557 males (34.58%) and 2,946 females (65.42%) participated in this study. The smoking rate was 17.57%, the drinking rate was 19.56%, and 38.53% said they did not exercise regularly. There were 2,247 overweight participants, accounting for 49.90% of all. In addition, we collected the dietary behaviors and found that only 1,623 (36.04%) of the participants could eat enough vegetables and fruits every day. During the follow-up period, there were 22 new stroke patients. The differences between stroke patients and controls are shown in Table 2. There was a significant gender difference between the two groups, with more women (86.36% vs. 65.32%, χ^2^ = 4.29, *p* < 0.05) among stroke patients. Participants who were physically inactive (59.09% vs. 38.43%, χ^2^ = 3.95, *p* < 0.05) had a greater chance of stroke. Stroke patients showed higher scores for BMI (26.09 ± 3.16 vs. 24.45 ± 3.35, *t* = −2.23, *p* < 0.05), CVAI (113.00 ± 26.71 vs. 91.46 ± 34.24, *t* = −2.95, *p* < 0.05), and waist-height ratio (0.55 ± 0.07 vs. 0.50 ± 0.06, *t* = −3.81, *p* < 0.05) compared with healthy participants.

**Table 1:** Basic information on each participant in this study.

Basic information	Participants (*n* = 4,503)
Community (Xinghai/Bailian)	2768/1735
Age (years)	57.86 ± 9.81
Gender (male/female)	1557/2946

**Table 2:** Demographic and clinical data of the study population.

Demographic and clinical data	Stroke patients (*n* = 22)	Controls (*n* = 4,481)	*t*/χ^2^/z	*P*-value
Age (years)	60.00 ± 9.10	57.85 ± 9.82	*t* = −1.03	0.31
Gender (male/female)	3/19	1554/2927	χ^2^ = 4.29	0.04^**^
Smoking (yes/never/quit)	3/19/0	788/3599/94	z = 0.17	0.86
Alcohol consumption (never/heavier drinking/light drinking)	19/2/1	3603/719/159	z = 0.68	0.50
Exercise (often/absent)	9/13	2759/1722	χ^2^ = 3.95	4.70×10^–2**^
Taste (salty/oily/sweet)	2/7/13	594/1119/2768	χ^2^ = 0.72	0.70
Meat and vegetarian (balanced/more meat/vegetarian based)	0/8/14	323/887/3271	z = 1.58	0.11^*^
6 taels of vegetables per day (basically every day/week<=2 days/other)	14/6/2	2539/1224/718	χ^2^ = 0.85	0.66
4 fruits per day (basically daily/week<=2 days/other)	7/8/7	1711/1386/1384	χ^2^ = 0.45	0.80
Family history of stroke (yes/no/unknown)	1/19/2	293/3915/273	z = 0.28	0.78
Family history of CHD (yes/no/unknown)	0/20/2	164/4041/276	z = 0.37	0.71
Family history of hypertension (yes/no/unknown)	6/14/2	1523/2671/287	χ^2^ = 0.60	0.74
Family history of diabetes (yes/no/unknown)	3/17/2	464/3737/280	z = 0.68	0.50
History of cerebrovascular disease (yes/no)	0/22	72/4409	z < 0.01	1.00
History of heart disease (yes/no)	0/22	149/4332	z < 0.01	1.00
Frequency of blood pressure measurement (never/often/occasionally)	2/5/15	496/1427/2558	χ^2^ = 1.12	0.57
History of hypertension (yes/no)	9/13	1348/3133	χ^2^ = 1.22	0.27
Frequency of lipid measurements (never/regular/occasional)	5/3/14	685/1148/2648	χ^2^ = 2.10	0.35
History of dyslipidemia (yes/no)	0/22	365/4116	χ^2^ = 1.01	0.31
Blood glucose measurement frequency (never/regular/occasional)	4/6/12	640/1229/2612	χ^2^ = 0.29	0.87
History of diabetes (yes/no)	3/19	349/4132	χ^2^ = 0.39	0.53
BMI	26.09 ± 3.16	24.45 ± 3.35	*t* = −2.23	0.02^**^
Waist circumference	85.91 ± 8.72	80.30 ± 9.07	*t* = −2.90	3.80×10^–3**^
SBP	135.80 ± 17.98	131.20 ± 14.45	*t* = −1.50	0.13^*^
DBP	83.11 ± 8.25	81.19 ± 8.89	*t* = −1.01	0.31
Heart murmur (yes/no)	0/22	9/4472	z < 0.01	1.00
Arrhythmia (no/yes)	22/0	4399/82	z < 0.01	1.00
Fasting blood glucose (mmol/L)	5.22 ± 1.10	5.31 ± 1.23	*t* = 0.34	0.73
HbA1c (%)	5.41 ± 0.44	5.53 ± 0.96	*t* = 1.30	0.21
Total cholesterol (mmol/L)	4.82 ± 0.88	4.71 ± 1.09	*t* = −0.43	0.66
HDL-C (mmol/L)	1.34 ± 0.28	1.36 ± 0.38	*t* = 0.31	0.76
LDL-C (mmol/L)	2.76 ± 0.81	2.65 ± 0.96	*t* = −0.54	0.59
Triglyceride (mmol/L)	1.58 ± 0.64	1.55 ± 0.96	*t* = −0.21	0.84
Homocysteine	8.54 ± 8.27	9.68 ± 6.62	*t* = 0.80	0.42
VAI	2.31 ± 1.28	2.08 ± 1.78	*t* = −0.60	0.55
CVAI	113.00 ± 26.71	91.46 ± 34.24	*t* = −2.95	3.20×10^–3**^
Triglyceride-glucose index	8.70 ± 0.50	8.63 ± 0.57	*t* = −0.58	0.57
Waist-height ratio	0.55 ± 0.07	0.50 ± 0.06	*t* = −3.81	1.41×10^–4**^

Continuous variables were expressed as mean ± SD and were statistically examined using two independent samples *T*-test. Categorical data were statistically examined using Chi-square test (χ^2^ and *P*-value) and Fisher’s exact test (z and *P*-value). CHD, coronary heart disease; SBP, systolic blood pressure; DBP, diastolic blood pressure; HbA1c, glycosylated hemoglobin; HDL-C, high-density lipoprotein cholesterol; LDL-C, low-density lipoprotein cholesterol; VAI, visceral adiposity index; CVAI, Chinese visceral adiposity index.

* *P* < 0.15.

** *P* < 0.05.

### Stroke risk factors and predictive model

We selected variables with *p* < 0.15 in univariate analysis and performed multivariate analysis. The age, gender, exercise, meat and vegetarian diet, BMI, waist circumference, systolic blood pressure, CVAI, and waist-height ratio were imported into the binary logistic regression model, and a stroke risk prediction scale was constructed. The results are shown in Table 3. Multivariate analysis found that gender (OR = 5.11, χ^2^ = 4.45, *p* = 0.04) and exercise (OR = 2.64, χ^2^ = 4.76, *p* = 0.03) were significant predictors of stroke.

**Table 3:** Multivariate logistic regression model for predicting stroke.

Variables	Odds ratio	95% CI	χ^2^	*P*-value
Age	1.03	0.96, 1.09	0.60	0.44
Gender	5.11	1.12, 23.27	4.45	0.04^*^
Exercise	2.64	1.10, 6.30	4.76	0.03^*^
Meat and vegetarian (balanced)	1.00			
Meat and vegetarian (more meat)	>999.99	<0.01, >999.99	3.00×10^−3^	0.96
Meat and vegetarian (vegetarian based)	>999.99	<0.01, >999.99	2.60×10^−3^	0.96
BMI	1.08	0.88, 1.32	0.51	0.47
Waist circumference	1.05	0.93, 1.19	0.65	0.42
SBP	1.01	0.96, 1.04	0.77	0.38
CVAI	1.00	0.97, 1.03	1.10×10^−3^	0.97
Waist-height ratio×100	0.99	0.82, 1.19	0.02	0.90

* *P* < 0.05.

SBP, systolic blood pressure; CVAI, Chinese visceral adiposity index.

### Predictive models for the overweight population

Stroke risk prediction scales were constructed for populations of different body types, and the results are shown in Table 4. We selected variables with *p* < 0.05 in univariate analysis and performed multivariate analysis. The stroke risk prediction scale for overweight population included age, gender, waist circumference, HbA1c, homocysteine, CVAI, and waist-height ratio. Homocysteine (OR = 0.82, χ^2^ = 4.95, *p* = 0.03) was a significant predictor of stroke in overweight individuals. The prediction scale for non-overweight population was composed of age, gender, family history of diabetes, BMI, waist circumference, systolic blood pressure, CVAI, and waist-height ratio.

**Table 4:** Multivariate logistic regression model for predicting stroke in overweight population and non-overweight population.

Variables	Odds ratio	95% CI	χ^2^	*P*-value
**Overweight**				
Age	1.01	0.94, 1.08	0.02	0.89
Gender	3.81	0.74, 19.74	2.54	0.11
Waist circumference	0.99	0.90, 1.10	0.02	0.90
HbA1c	0.59	0.28, 1.24	1.94	0.16
Homocysteine	0.82	0.69, 0.98	4.95	0.03^*^
CVAI	1.01	0.98, 1.04	0.24	0.62
Waist-height ratio×100	1.07	0.92, 1.25	0.77	0.38
**Non-overweight**				
Age	1.03	0.93, 1.15	0.29	0.59
Gender	6.08	0.39, 94.62	1.66	0.20
Family history of diabetes (yes)	1.00			
Family history of diabetes (no)	0.23	0.04, 1.25	2.89	0.09
Family history of diabetes (unknown)	0.38	0.03, 4.56	0.58	0.45
BMI	1.43	0.66, 3.10	0.81	0.37
Waist circumference	1.17	0.87, 1.59	1.05	0.31
Systolic blood pressure	1.04	0.99, 1.08	2.72	0.10
CVAI	1.01	0.95, 1.06	0.03	0.87
Waist-height ratio×100	0.87	0.55, 1.38	0.37	0.55

* *P* < 0.05.

HbA1c, glycosylated hemoglobin; CVAI, Chinese visceral adiposity index.

### Performance of predictive models

According to the ROC analysis of the prediction model for total community-dwelling population, the high-risk group was classified based on the optimal critical point. There were significant differences between high-risk group and low-risk group in various aspects (*p* < 0.05), such as age, gender, lifestyle behaviors, and blood pressure, as shown in Supplementary Table I. The Youden index was 0.47. The accuracy of the prediction model was higher than the “8 + 2” risk score, which had the Youden index of 0.08. The McNemar’s test results showed that there was no significant difference in sensitivity, while the specificity of the prediction model was significantly higher than the “8 + 2” risk score (χ^2^ = 59.26, *p* < 0.05).

**Figure 1:**
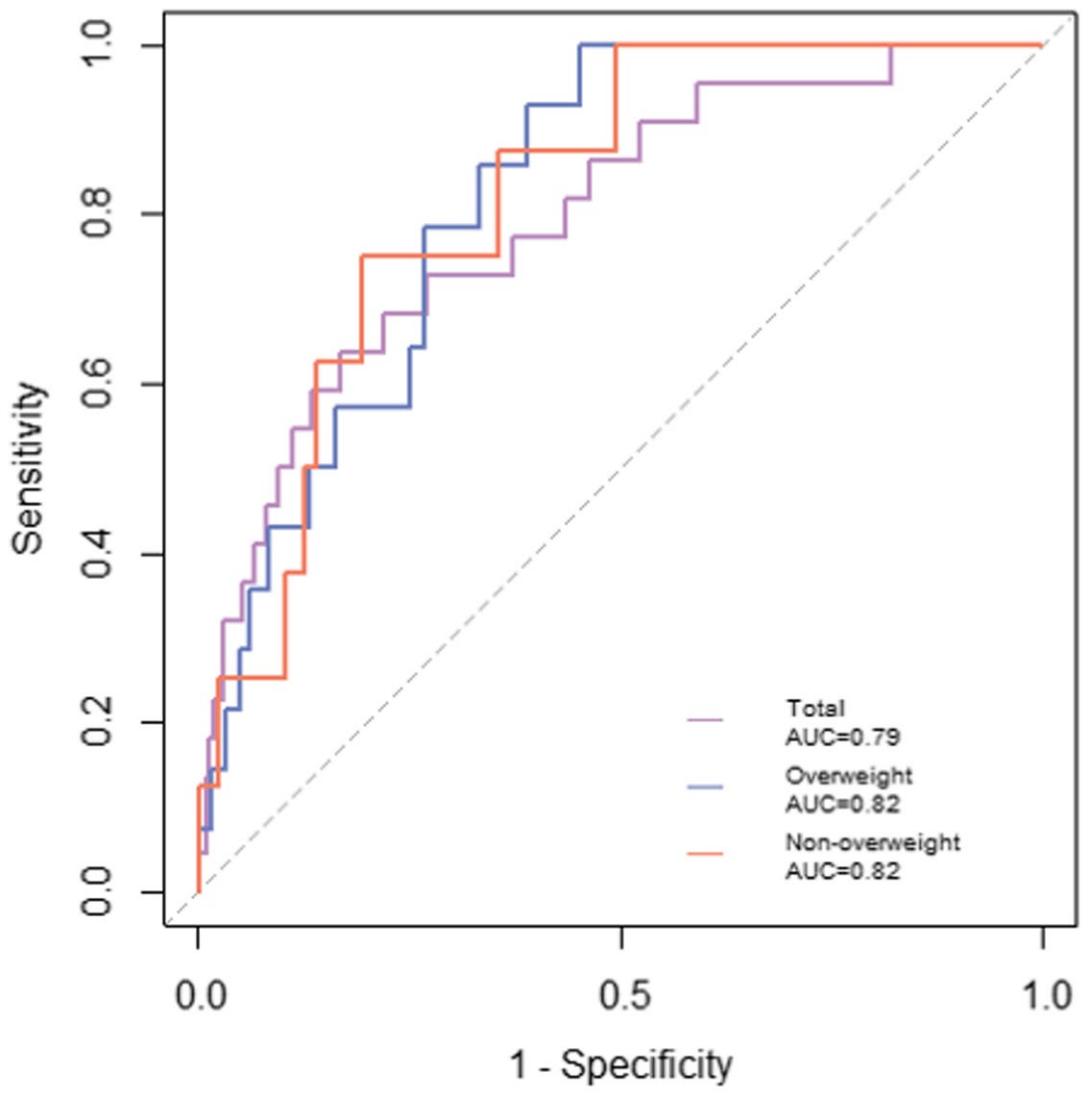
ROC curves generated for predicting stroke. The stroke prediction model for the total population was composed of age, gender, exercise, meat and vegetarian diet, BMI, waist circumference, systolic blood pressure, CVAI, and waist-height ratio, and the AUC was 0.79, with *p* < 0.05. Prediction model for overweight population composed of age, gender, waist circumference, HbA1c, homocysteine, CVAI, and waist-height ratio; age, gender, family history of diabetes, BMI, waist circumference, systolic blood pressure, CVAI, and waist-height ratio constituted the prediction model for non-overweight population, and the AUCs were both 0.82, with *p* < 0.05.

The accuracy of the stroke risk prediction models was demonstrated by ROC analysis, as shown in Figure 1. The stroke prediction scale composed of age, gender, exercise, meat and vegetable diet, BMI, waist circumference, systolic blood pressure, CVAI, and waist-height ratio, showed high prediction accuracy. The area under the ROC curve (AUC) was 0.79 (z = 4.76, *p* < 0.05). In addition, the figure also showed the ROC curves of the two subgroup prediction scales. The overweight population prediction scale and the non-overweight population prediction scale had similar performance, with the AUCs of 0.82 (z = 4.14, *p* < 0.05) and 0.82 (z = 3.12, *p* < 0.05). The results indicated that three prediction scales could effectively and accurately predict the 2-year stroke risk in the community-dwelling population.

**Figure 2:**
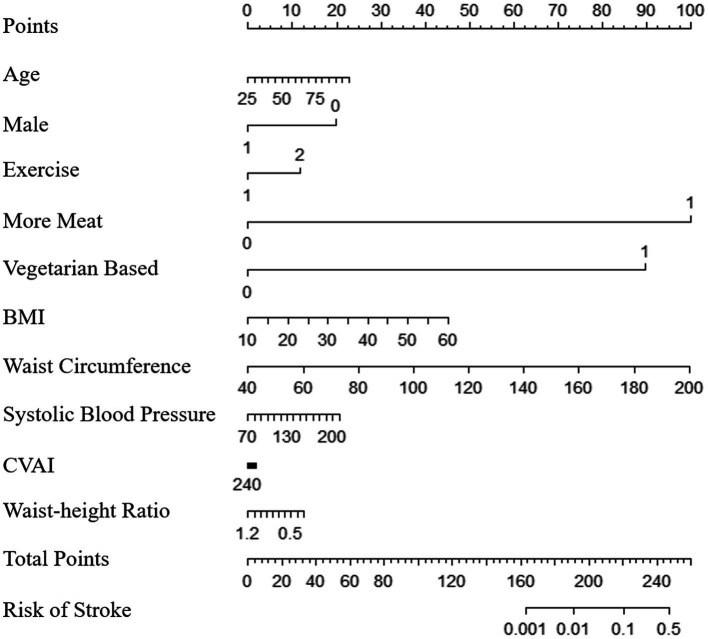
Nomogram for predicting stroke in community-dwelling population. The nomogram could be used by first locating the position of each variable on the corresponding axis. The number of points for each variable is found on the point axis. The sum of all points is the total points, and the risk of stroke is determined by drawing a vertical downward line from the total point axis.

After constructing and validating the predictive models, we established nomograms (Figure 2; Supplementary Figure II) and an online computing tool based on them, which helped doctors intelligently obtain the 2-year stroke risk of the community-dwelling persons.

## Discussion

In this study, a high-accuracy stroke prediction model was constructed, consisting of age, gender, exercise, meat and vegetarian diet, BMI, waist circumference, systolic blood pressure, CVAI, and waist-height ratio. Two additional prediction models for overweight and non-overweight individuals were formulated based on a list of risk factors, respectively. These models identified factors affecting stroke in the community-dwelling population and could predict 2-year risk with high accuracy. Nomograms and online prediction system provided community doctors with optional tools to distinguish high-risk groups conveniently and accurately, and furnished evidence for early identification and treatment.

Regarding the relationship between gender and stroke, there were significant sexual differences in incidence, presentation, and prognosis, although the mechanism remained unclear (Saini and Shuaib, 2008). Our study provided evidence for the effect of gender on stroke incidence. Compared with men, women over the age of 40 had a 5.11-times higher risk of stroke. It was consistent with fluctuations in hormone levels of women, possibly related to the vascular protective properties of estrogen and the decrease of estrogen after menopause (Cordonnier et al., 2017). Women over the age of 40 should be considered the primary population for stroke prevention and management.

It was recognized that obese individuals were more likely to suffer from various chronic diseases. People who were overweight or obese had a higher risk of stroke, hypertension, diabetes, and other diseases (Global Burden of Metabolic Risk Factors for Chronic Diseases Collaboration et al., 2014; Bragg et al., 2018; Liu et al., 2020). Therefore, although BMI was not significant in the stroke prediction model for the total population in this study, we still divided the population into overweight and non-overweight groups to construct models, respectively. BMI, waist circumference, and waist-height ratio, as traditional body fat indicators, were widely used to predict cardiovascular and cerebrovascular diseases including stroke (Lam et al., 2015; Ohlsson et al., 2017). Our findings in the univariate analysis also suggested that stroke populations had higher scores of BMI, waist circumference, and waist-height ratio. CVAI was an evaluation index of visceral fat calculated based on the age, BMI, waist circumference, triglyceride, and HDL of the Chinese population. Compared with the traditional indicators, CVAI could better reflect the visceral fat content of the human body (Tsou et al., 2021). It had also been demonstrated a significant correlation with the causes of cardiovascular and cerebrovascular diseases (Xia et al., 2016; Han et al., 2021), but there were few studies on the correlation between CVAI and the risk of stroke. This study provided evidence for the association of CVAI with stroke, and used this novel indicator to predict the occurrence of stroke. The results confirmed that CVAI was significantly associated with stroke, with higher CVAI in stroke patients. However, this significant association was weakened after adjusting for other factors. Another novel evaluation index, VAI, showed no significant difference between stroke patients and controls.

Negative lifestyle behaviors increased the risk of stroke. Regular exercise affected the development of atherosclerosis and reduced the risk of cardiovascular events (Green and Smith, 2018). Highly physically active population had a 27% lower risk of stroke morbidity or mortality compared with the inactive population (Lee et al., 2003). Moreover, physical activity might affect stroke severity, and inactivity was associated with increased stroke severity (Reinholdsson et al., 2018). Our study indicated that lack of exercise increased the risk of stroke, which was consistent with previous studies. Individuals who lacked physical activity had a 2.64-times increased risk of stroke than those who exercised regularly. For the population at high risk of stroke, they should first realize the harm of lack of exercise, and then improve their exercise behavior through planning, monitoring, and feedback (Prior and Suskin, 2018). Community doctors should provide corresponding knowledge and exercise guidance. The fitness facilities and fitness trails in the community park also provide convenient conditions. Everyone could reduce the risk of stroke through regular physical activities, regardless of gender, age, and other vascular factors, reducing blood pressure and improving lipid status and vascular function (Garcia-Cabo and Lopez-Cancio, 2020). Previous research also attempted to identify the relationship between alcohol consumption and stroke. A prospective cohort study of more than 10,000 participants followed for 10 years found that different drinking patterns had different effects on stroke risk (Sundell et al., 2008). It suggested that heavy drinking was an independent risk factor for total and ischemic stroke. However, light drinking was associated with a lower risk of stroke, independent of the type of alcohol (Lu et al., 2008). Smoking had been recognized as a risk factor for various diseases (Maritz and Mutemwa, 2012). Studies in different regions and populations showed a strong correlation between smoking and stroke, with smokers having a significantly higher risk compared to nonsmokers or those who had quit smoking (Shah and Cole, 2010). Quitting smoking and reducing alcohol consumption may be beneficial options to reduce the risk of stroke: 1 year after quitting smoking, the risk will be reduced by 50%, and moderate drinking may reduce the risk of stroke by 30% (Sarikaya et al., 2015). Among our study participants, 9.09% and 4.55% of the stroke patients drank a lot and a little, and 13.64% of the stroke patients smoked. However, there was no significant difference compared with controls. The absence of significant effects in our study might result from the short follow-up time and small patient collectives. Dietary behaviors were also a valuable factor in preventing stroke, which had attracted attention recently. It had been demonstrated that a dietary pattern similar to the Mediterranean diet and reducing red meat intake could decrease stroke risk (Spence, 2018). A healthy plant-based diet was associated with a lower risk of stroke, while an unhealthy vegetarian diet did not reduce the risk of stroke (Baden et al., 2021). Previous research focused on the relationship between dietary patterns and stroke, but no research on detailed dietary behaviors. Our study confirmed the relationship between dietary behaviors and stroke, including taste, meat and vegetable diet, and vegetable and fruit intake, where the meat and vegetable diet was included in the logistic model for predicting stroke. For persons at risk of stroke, they are advised to eat a diet similar to the Mediterranean diet, that is, eat beneficial oils, grains, fruits, vegetables, and beans, and limit the intake of red meat and sodium; maintaining a balanced and healthy diet will greatly help prevent stroke and reduce the risk of cardiovascular and cerebrovascular diseases (Spence, 2018).

Previous studies strived to identify risk factors of stroke and build predictive models in various populations and regions. A prospective study in Japan constructed a stroke risk equation based on a cohort from multiple centers, including age, gender, smoking, systolic blood pressure, antihypertensive drug use, diabetes, and HDL-C (Yatsuya et al., 2016). The risk equation could estimate the 10-year probability of ischemic stroke, with the AUC of 0.78. This study had a large sample and a long follow-up time, but the dietary behaviors were not considered as the potential predictors. Julia et al. developed and validated a risk algorithm to assess the risk of stroke or transient ischemic attack in patients without stroke or transient ischemic attack at baseline. Predictors such as systolic blood pressure, blood lipid level, smoking, ethnicity, and several stroke-related disease histories were included (Hippisley-Cox et al., 2013). The subjects covered the population aged from 25 to 84, while our study built a stroke risk prediction model for the community-dwelling population with a higher age (over 40 years old). Zhang et al. followed up 4,400 male steel workers aged 18–74 for more than 10 years, and constructed risk scoring models for ischemic stroke and hemorrhagic stroke based on a Cox regression model, showing good predictive performance, with AUCs of 0.72 and 0.82, respectively, (Zhang et al., 2005). In terms of the applicable population of the model, it is obviously different from the stroke prediction model in this study. All these findings were inconsistent because of the limitations of sample size and information collected. Our study included the usual demographic characteristics and vascular risk factors, as well as some associated indicators that did not be covered by previous studies. We completed a more comprehensive analysis of risk factors and constructed a 2-year stroke prediction model based on age, gender, exercise, meat and vegetable diet, BMI, waist circumference, systolic blood pressure, CVAI, and waist-height ratio. We also constructed alternative predictive models for overweight and non-overweight populations.

Although previous studies had provided various stroke risk prediction models, there was still a lack of prediction models suitable for the population on a high-sugar diet. As a risk factor for stroke, the high-sugar diet was a typical dietary feature in Suzhou and other southeastern China regions, which had been proved the relevance with stroke risk factors (Ahmad et al., 2020). Higher intake of artificially sweetened soft drinks increased the risk of ischemic stroke (Pase et al., 2017). Therefore, it was necessary to construct a stroke risk prediction model for the high-sugar diet population. Based on the Suzhou community-dwelling population, our study analyzed the demographic and clinical characteristics of all participants, and built a scale that filled this gap.

Our study has some limitations. We did not distinguish between subtypes of stroke outcomes. We should identify the risk factors of ischemic and hemorrhagic stroke separately, comparing the lifestyle behaviors and clinical characteristics of patients with different subtypes of stroke. Predictors of ischemic stroke and hemorrhagic stroke may differ. In addition, we should follow the participants for a longer period. Since the mean follow-up period of the study was only 2 years, the number of patients with stroke was small. This was also the reason why we did not distinguish between stroke subtypes, which will reduce the accuracy of prediction. This study also lacked other cohorts for the validation. We will pay attention to these in future research.

## Conclusion

In conclusion, this longitudinal cohort study explored stroke risk factors based on large sample size. The 2-year stroke prediction models were constructed based on lifestyle behaviors and clinical characteristics. The nomograms and online prediction system established on this basis could predict the risk of stroke for community-dwelling population over 40 years old. These provide convenient tools for accurately identifying those who are at high risk and require early treatment. Individuals with high risk should consider whether they will change lifestyle behaviors and receive medication. The results of this study provide guidance for community disease management, and are of great significance for improving prevention and treatment strategies of stroke.

## Data availability statement

The datasets presented in this article are not readily available because of the ethical and privacy restrictions. Requests to access the datasets should be directed to YY, yyao@fudan.edu.cn.

## Ethics statement

The studies involving human participants were reviewed and approved by the Ethics Committee of First Affiliated Hospital of Soochow University. The patients/participants provided their written informed consent to participate in this study.

## Author contributions

XT and YY designed the study. LZ, YL, XT, and QF recruited participants and collected the data. QW and YY analyzed related statistics. QW, LZ, and YY wrote the manuscript. LZ, YL, XT, YY, and QF supervised the study and revised the manuscript. All authors contributed to the article and approved the submitted version.

## Funding

This study was supported by the National Natural Science Foundation of China (no. 82001125 to XT) and Natural Science Foundation of Jiangsu Province (no. BK20180201 to XT).

## Conflict of interest

The authors declare that the research was conducted in the absence of any commercial or financial relationships that could be construed as a potential conflict of interest.

## Publisher’s note

All claims expressed in this article are solely those of the authors and do not necessarily represent those of their affiliated organizations, or those of the publisher, the editors and the reviewers. Any product that may be evaluated in this article, or claim that may be made by its manufacturer, is not guaranteed or endorsed by the publisher.
