# Supplementary material for: Development of stroke predictive model in community-dwelling population: A longitudinal cohort study in Southeast China
Source: Front Aging Neurosci. 2022 Dec 22;14:1036215. doi: 10.3389/fnagi.2022.1036215 (PMC9813513; doi:10.3389/fnagi.2022.1036215)
Supplement: Supplementary file 1 [file Data_Sheet_1.docx]

Supplementary Material

**Table****Ⅰ** The difference between high-risk population and low-risk population.

| Demographic and clinical data | high-risk population  (n = 772) | low-risk population  (n = 3731) | *t* / χ^2^ / z | *P*-value |
| --- | --- | --- | --- | --- |
| Age (years) | 60.94 ± 9.99 | 57.22 ± 9.66 | *t* = -9.76 | 2.78$\times$10^-22*^ |
| Gender (male/female) | 45/727 | 1512/2219 | χ^2^ = 240.40 | 3.22$\times$10^-54*^ |
| Smoking (yes/never/quit) | 16/755/1 | 775/2863/93 | χ^2^ = 179.76 | 9.24$\times$10^-40*^ |
| Alcohol consumption (never/heavier Drinking/light drinking) | 750/12/10 | 2872/709/150 | χ^2^ = 167.36 | 4.55$\times$10^-37*^ |
| Exercise (often/absent) | 216/556 | 2552/1179 | χ^2^ = 441.25 | 5.79$\times$10^-98*^ |
| Taste (salty/oily/sweet) | 94/285/393 | 502/841/2388 | χ^2^ = 71.43 | 3.08$\times$10^-16*^ |
| Meat and vegetarian (balanced/more meat/vegetarian based) | 0/326/446 | 323/569/2839 | χ^2^ = 330.48 | 1.73$\times$10^-72*^ |
| 6 taels of vegetables per day (basically every day/week<=2 days/other) | 417/189/166 | 2136/1041/554 | χ^2^ = 21.63 | 2.01$\times$10^-5*^ |
| 4 fruits per day (basically daily/week<=2 days/other) | 226/233/313 | 1492/1161/1078 | χ^2^ = 47.54 | 4.75$\times$10^-11*^ |
| Family history of stroke (yes/no/unknown) | 55/682/35 | 239/3252/240 | χ^2^ = 4.38 | 0.11 |
| Family history of CHD (yes/no/unknown) | 27/710/35 | 137/3351/243 | χ^2^ = 4.44 | 0.12 |
| Family history of hypertension (yes/no/unknown) | 266/469/37 | 1263/2216/252 | χ^2^ = 4.10 | 0.13 |
| Family history of diabetes (yes/no/unknown) | 83/655/34 | 384/3099/248 | χ^2^ = 5.51 | 0.06 |
| History of cerebrovascular disease (yes/no) | 15/757 | 57/3674 | χ^2^ = 0.70 | 0.40 |
| History of heart disease (yes/no) | 30/742 | 119/3612 | χ^2^ = 0.97 | 0.32 |
| Frequency of blood pressure measurement (never/often/occasionally) | 62/237/473 | 436/1195/2100 | χ^2^ = 10.86 | 4.38$\times$10^-3*^ |
| History of hypertension (yes/no) | 348/424 | 1009/2722 | χ^2^ = 98.81 | 2.78$\times$10^-23*^ |
| Frequency of lipid measurements (never/regular/occasional) | 100/152/520 | 590/999/2142 | χ^2^ = 26.69 | 1.60$\times$10^-6*^ |
| History of dyslipidemia (yes/no) | 70/702 | 295/3436 | χ^2^ = 1.16 | 0.28 |
| Blood glucose measurement frequency (never/regular/occasional) | 95/188/489 | 549/1047/2135 | χ^2^ = 9.91 | 0.01^*^ |
| History of diabetes (yes/no) | 97/675 | 255/3476 | χ^2^ = 29.15 | 6.70$\times$10^-8*^ |
| BMI | 26.70 ± 3.83 | 23.99 ± 3.05 | *t* = -18.48 | 1.48$\times$10^-73*^ |
| Waist circumference | 85.84 ± 9.59 | 79.18 ± 8.53 | *t* = -17.89 | 3.34$\times$10^-69*^ |
| SBP | 137.60 ± 16.35 | 129.90 ± 13.69 | *t* = -12.16 | 1.69$\times$10^-33*^ |
| DBP | 82.59 ± 9.43 | 80.91 ± 8.74 | *t* = -4.56 | 5.25$\times$10^-6*^ |
| Heart murmur (yes/no) | 2/770 | 7/3724 | χ^2^ < 0.01 | 1.00 |
| Arrhythmia (no/yes) | 750/22 | 3671/60 | χ^2^ = 5.52 | 0.02^*^ |
| Fasting blood glucose (mmol/L) | 5.41 ± 1.27 | 5.29 ± 1.22 | *t* = -2.55 | 0.01^*^ |
| HbA1c (%) | 5.63 ± 1.02 | 5.51 ± 0.94 | *t* = -3.10 | 1.95$\times$10^-3*^ |
| Total cholesterol (mmol/L) | 4.95 ± 1.16 | 4.67 ± 1.07 | *t* = -6.33 | 2.69$\times$10^-10*^ |
| HDL-C (mmol/L) | 11.38 ± 0.35 | 1.36 ± 0.39 | *t* = -1.36 | 0.17 |
| LDL-C (mmol/L) | 2.81 ± 1.01 | 2.62 ± 0.94 | *t* = -4.86 | 1.22$\times$10^-6*^ |
| Triglyceride (mmol/L) | 1.69 ± 0.95 | 1.52 ± 0.96 | *t* = -4.40 | 1.11$\times$10^-5*^ |
| Homocysteine | 9.59 ± 6.03 | 9.69 ± 6.75 | *t* = 0.40 | 0.69 |
| VAI | 2.48 ± 1.81 | 2.00 ± 1.75 | *t* = -6.84 | 8.98$\times$10^-12*^ |
| CVAI | 118.50 ± 29.64 | 85.99 ± 32.44 | *t* = -27.29 | 6.99$\times$10^-152*^ |
| Triglyceride-glucose index | 8.75 ± 0.54 | 8.60 ± 0.57 | *t* = -6.68 | 2.68$\times$10^-11*^ |
| Waist-height ratio | 0.55 ± 0.07 | 0.49 ± 0.05 | *t* = -23.98 | 1.08$\times$10^-119*^ |

Continuous variables were expressed as mean ± SD and were statistically examined using two independent samples *T*-test. Categorical data were statistically examined using Chi-square test (χ^2^ and *P*-value) and Fisher’s exact test (z and *P*-value). ^*^*P* < 0.05.

Abbreviation: CHD: coronary heart disease, SBP: systolic blood pressure, DBP: diastolic blood pressure, HbA1c: glycosylated hemoglobin, HDL-C: high-density lipoprotein cholesterol, LDL-C: low-density lipoprotein cholesterol, VAI: visceral adiposity index, CVAI: Chinese visceral adiposity index.


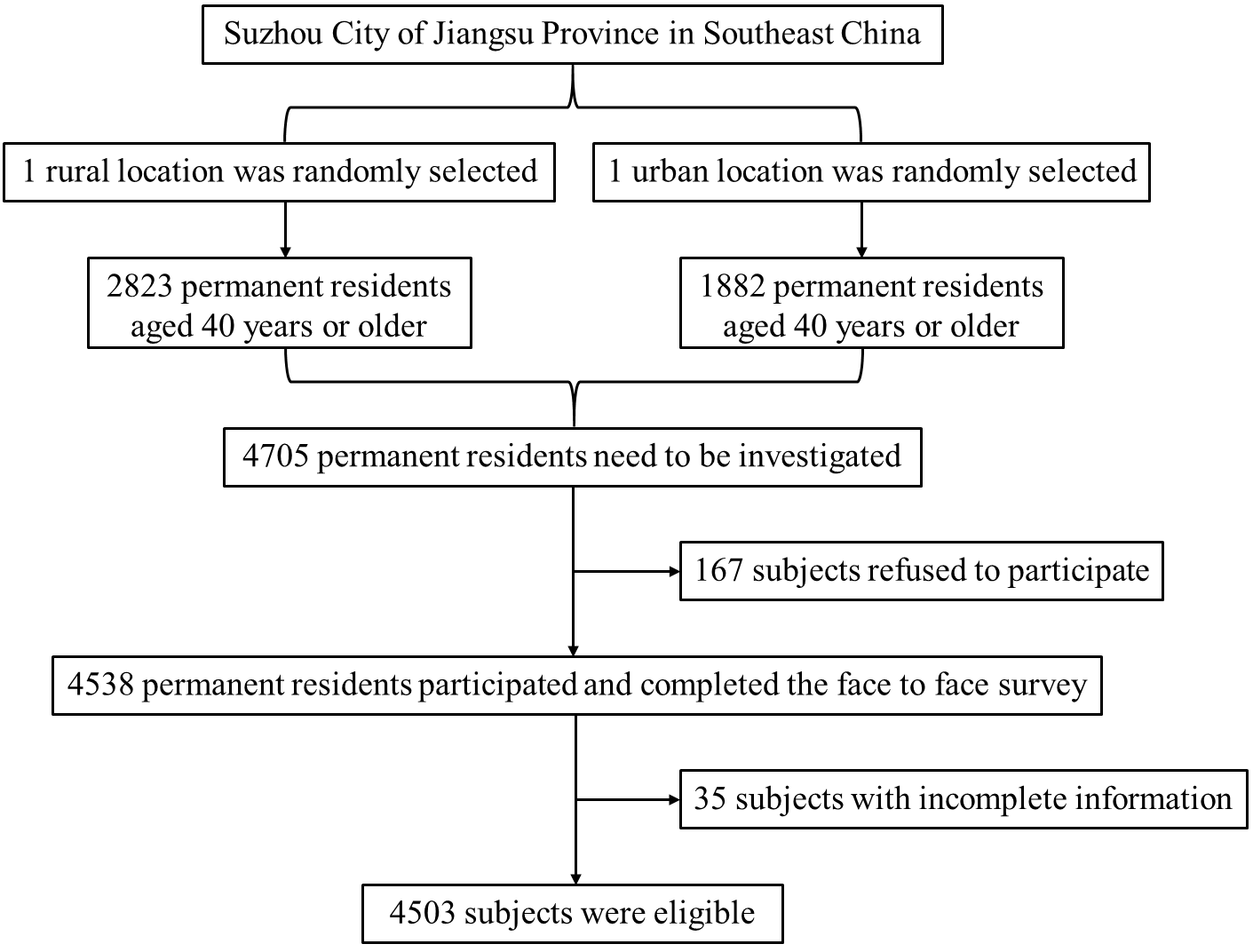
**FigureⅠ** Flowchart of the enrollment of subjects**.**


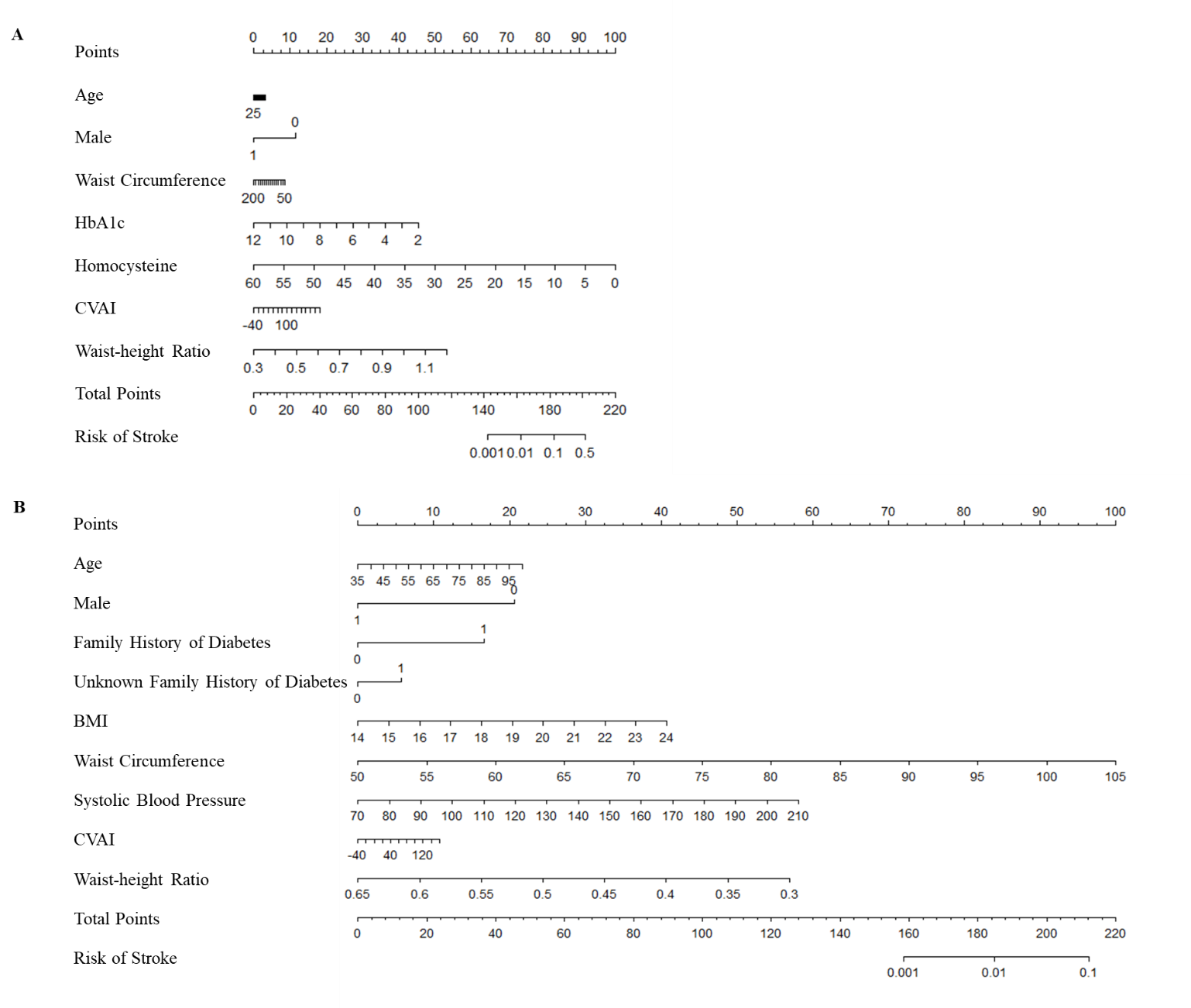


**Figure Ⅱ** Nomograms for predicting stroke in overweight population (A) and non-overweight population (B).
